# Therapy-resistant acute lymphoblastic leukemia (ALL) cells inactivate FOXO3 to escape apoptosis induction by TRAIL and Noxa

**DOI:** 10.18632/oncotarget.953

**Published:** 2013-06-25

**Authors:** Michael J. Ausserlechner, Christina Salvador, Andrea Deutschmann, Martin Bodner, Giampietro Viola, Roberta Bortolozzi, Giuseppe Basso, Judith Hagenbuchner, Petra Obexer

**Affiliations:** ^1^ Department of Pediatrics I, Medical University Innsbruck, Austria; ^2^ Department of Pediatrics II, Medical University Innsbruck, Austria; ^3^ Tyrolean Cancer Research Institute, Innsbruck, Austria; ^4^ Department of Woman's and Child's Health, Oncohematology laboratory University of Padova, Padova-Italy; ^5^ present affiliation: Institute of Legal Medicine, Innsbruck Medical University, Innsbruck, Austria

**Keywords:** FOXO3/FKHRL1, T-ALL, p16/INK4A, BH3-only proteins, TRAIL

## Abstract

Forkhead transcription factors (FOXO) are downstream targets of the phosphoinositol-3-kinase (PI3K) protein kinase B (PKB) signaling cascade and play a pivotal role in cell differentiation, cell cycle and apoptosis. We found that cells from prednisone-resistant T-acute lymphoblastic leukemia (T-ALL) patients showed cytoplasmic localization of FOXO3 in comparison to prednisone-sensitive patients suggesting its inactivation. To determine the impact of FOXO3, T-ALL cells were infected with a 4OH-tamoxifen-regulated, phosphorylation-independent FOXO3(A3)ERtm allele. After FOXO3-activation these cells undergo caspase-dependent apoptosis. FOXO3 induces the death ligand TRAIL and the BH3-only protein Noxa implicating extrinsic as well as intrinsic death signaling. Whereas dnFADD partially inhibited cell death, CrmA and dnBID efficiently rescued ALL cells after FOXO3 activation, suggesting a caspase-8 amplifying feedback loop downstream of FADD. Knockdown of TRAIL and Noxa reduced FOXO3-induced apoptosis, implicating that mitochondrial destabilization amplifies TRAIL-signaling. The-reconstitution of the cell cycle inhibitor p16^INK4A^, which sensitizes ALL cells to mitochondria-induced cell death, represses FOXO3 protein levels and reduces the dependency of these leukemia cells on PI3K-PKB signaling. This suggests that if p16^INK4A^ is deleted during leukemia development, FOXO3 levels elevate and FOXO3 has to be inactivated by deregulation of the PI3K-PKB pathway to prevent FOXO3-induced cell death.

## INTRODUCTION

Forkhead transcription factors (FOXO) are downstream targets of the phosphoinositol-3-kinase (PI3K) protein kinase B-(PKB)-signaling pathway [1]. The FOXO subfamily consists of four members (FOXO1, FOXO3, FOXO4, and FOXO6), which regulate cell type specific apoptosis, cell cycle arrest or stress responses [2]. Due to deregulation of the PI3K-PKB pathway, these transcription factors are inactivated in the majority of human malignancies, either through hyperactivation of PKB itself or due to the loss of inhibitors of the PI3K-signaling, such as PTEN [3]. In 50-75% of T-acute lymphoblastic leukemia (T-ALL) patients a hyperactivation of the PI3K-PKB pathway is found, which is predictive for a poor clinical outcome [4]. The reasons for PKB activation are not completely clarified as neither mutations of the PI3K or the PKB are associated with ALL nor mutations of tyrosine kinases like BRC-ABL or FLT3-ITD are relevant for PKB activation in leukemia [5].

One explanation for PKB hyperactivation might be that T-ALL cells frequently carry a heterozygous deletion of the PTEN gene locus [6], which leads to increased PI3K-PKB signaling and thereby inactivation of the PKB target FOXO3 in leukemia cells. Inactivation of the PTEN locus correlates with increased proliferation and replicative senescence [7], leukemogenesis [8] and predicts relapse in childhood ALL [9]. Inhibitors of the PI3K-PKB pathway, which induce apoptosis in different leukemia cells are already in the focus of several preclinical studies but the critical target in this network has not been discovered yet [4, 10].

FOXO3 was shown to play a crucial role in controlling cell cycle arrest, apoptosis and self-renewal of haematopoietic progenitor cells [11, 12] and to act as a barrier to hematopoietic transformation which is overcome by activation of PI3K/PKB signaling [13-15]. Deletion of FOXO family members in mice leads to the development of T-cell lymphoma, demonstrating that the PKB-FOXO axis is involved in tumor development [16]. Active FOXO3, however, regulates apoptosis signaling cell type specific by induction of the BH3-only proteins PMAIP1/Noxa and BCL2L11/Bim [17, 18], or by induction of the death ligands TRAIL or FASL [1, 19]. FOXO3 indirectly interferes with the balance of pro-and anti-apoptotic BCL2 proteins by the induction of B-cell lymphoma 6 (Bcl6), which represses the anti-apoptotic protein BCL2L1/BclxL [20].

Apoptosis is initiated either by binding of so called death ligands, such as FASL or TRAIL to their cognate receptors resulting in formation of the death-inducing signaling complex (DISC) and activation of CASP8/caspase-8, or via the intrinsic death pathway. The intrinsic death pathway is controlled at the level of mitochondria by members of the BCL2 family. This protein family is divided into anti-apoptotic members, such as BCL2, BclxL or MCL1, the pro-apoptotic BH3-only proteins (such as Noxa, Bim or BBC3/Puma) and the pro-apoptotic multi-domain proteins BAX, BAK and Bok. The balance of pro- and anti-apoptotic proteins (BCL2 rheostat) regulates mitochondrial membrane potential. If damaged, Cytochrome *c* is released from mitochondria and triggers the formation of the apoptosome, which leads to subsequent activation of CASP9/caspase-9. Two models have been discussed how BH3-only proteins induce cell death. The first model describes activators of BAX and BAK, like Bid and Bim, and sensitizers like Noxa which bind to anti-apoptotic proteins and thereby release activator BH3-only proteins as well as BAX and BAK [21]. The second model implies that the main function of the anti-apoptotic BCL2 proteins is to sequester BAX and BAK and to prevent their insertion into the mitochondrial membrane. BH3-only proteins thereby displace BAX and BAK by binding with different affinity to BCL2 proteins.

The BH3-only protein BID connects the extrinsic and intrinsic signaling, because it is cleaved by active caspase-8 and then inserts into the outer mitochondrial membrane where it antagonizes the function of the pro-survival BCL2 proteins. In some cell types (so called type II cells) extrinsic death signaling always involves amplification of the death signal via mitochondria, since overexpression of either BCL2 or BclxL prevents TRAIL-induced apoptosis in CEM cells [22]. This is caused by reduced DISC formation in type II cells compared to type I cells, were extrinsic signaling directly activates CASP3/caspase-3, independent of mitochondrial involvement.

In this study we investigated whether therapy resistance in childhood T-ALL cells correlates with inactivation of FOXO3. We uncovered, that FOXO3 activates apoptosis by induction of TRAIL and Noxa and found that the expression of the frequently mutated tumor suppressor p16^INK4A^ in T-ALL represses endogenous FOXO3, suggesting that these two tumor suppressor proteins cooperate to prevent childhood leukemia.

## RESULTS

### Cellular FOXO3-localization correlates with a therapy-resistant T-ALL phenotype

**Figure 1:**
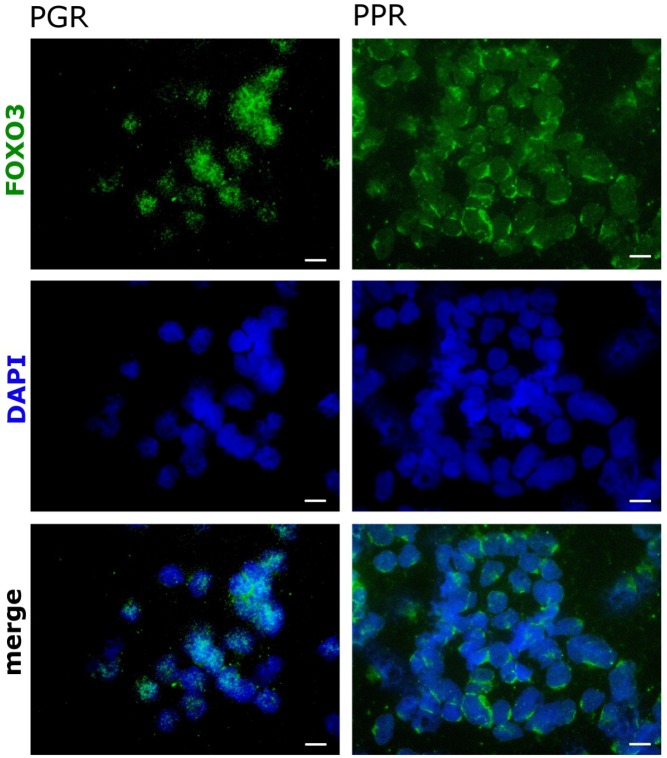
FOXO3 localizes to the cytoplasm in bone marrow cells from prednisone-resistant T-ALL pediatric patients Bone marrow cells from pediatric T-ALL patients sensitive (PGR) or resistant (PPR) to prednisone were fixed before treatment in 4% formaldehyde. FOXO3 expression was visualized with a specific rabbit monoclonal antibody against FOXO3, followed by the addition of ALEXA488 (green) labeled anti-rabbit antisera. DAPI (blue) was applied to visualize the nuclei. Images were acquired by the videoconfocal system ViCo microscope Nikon Eclipse 80i (Nikon, Japan). The shown stainings are representative for three PGR and three PPR patients.

Deregulation of the PI3K/PKB/FOXO3 pathway was shown to be involved in cancer development and contributes to therapy resistance of different malignancies. Bone marrow cells from pediatric T-ALL patient samples were divided into good responders to initial prednisone therapy (PGR) and prednisone poor responders (PPR) and were analyzed by immunofluorescence for FOXO3 expression and subcellular localization. As shown in Fig 1, cells from PGR patients demonstrate a predominantly nuclear localization of FOXO3 in comparison to PPR. This partial activation of FOXO3 might sensitize these cells to further, apoptosis-inducing therapies and thereby contribute to a positive therapy response.

### Ectopic FOXO3 induces Caspase-dependent cell death in T-ALL

**Figure 2:**
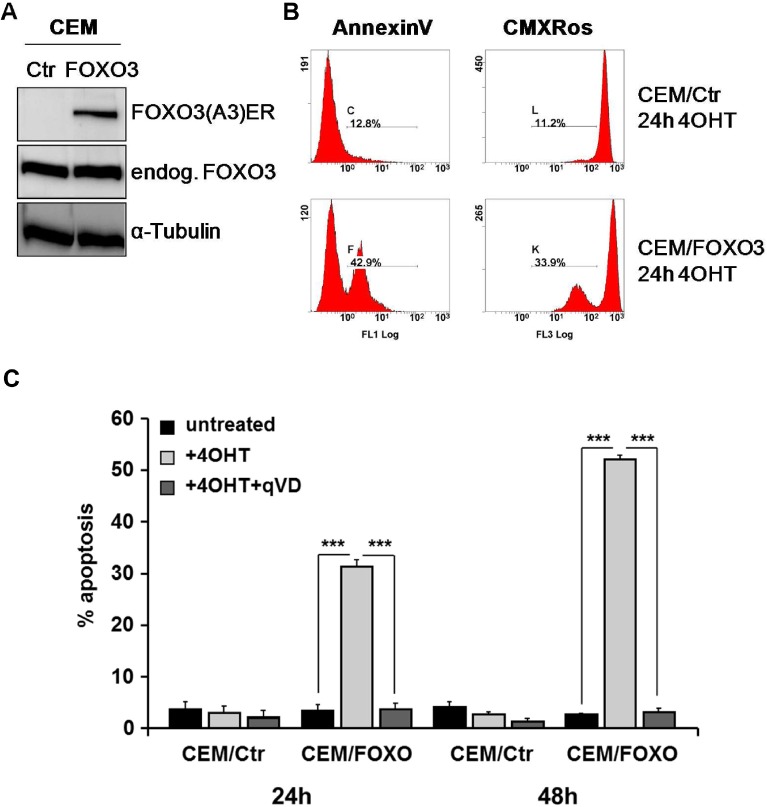
FOXO3 activation induces caspase-dependent apoptotic cell death in CEM cells The vector pLIB-FOXO3(A3)ERtm-iresNeo was retrovirally infected into CEM cells. Expression of the endogenous FOXO3 and the fusion protein FOXO3(A3)ERtm in CEM/FOXO3 cells were assessed by immunoblot analysis. α-Tubulin served as loading control. Mock-transfected CEM/Ctr cells were used as control (A, left panel). CEM/FOXO3 cells were incubated with 4OHT (50 nM) for 24 hours to activate transgenic FOXO3. Exposure of phosphatidylserin was analyzed by AnnexinV staining and loss of mitochondrial activity was detected by CMXRos staining (B). CEM/Ctr and CEM/FOXO3 cells were treated with 4OHT (50 nM) and/or the caspase inhibitor qVD (10 µM) for 24 and 48 hours. Apoptosis was measured by PI-FACS analyses (C). Statistical difference between treatments was assessed by unpaired t-test (***P < 0.001).

As FOXO3 activation leads to apoptosis induction in haematopoietic cells [11, 12], we investigated whether FOXO3 inactivation in PPR ALL cells may account for therapy resistance and apoptosis inhibition. To analyze the function of FOXO3 in ALL cells, we infected different T-ALL-cells lines (CEM, Jurkat, Molt3 and Molt4) with a retrovirus coding for a PKB-phosphorylation-independent, estrogen receptor FOXO3(A3)ERtm fusion protein. The expression of the fusion protein was verified by immunoblot (Fig 2A and supplemental Fig. 1A). Activation of FOXO3 by treatment with 4-OH-tamoxifen (4OHT) in CEM/FOXO3 cells for 24 hours increases the number of AnnexinV positive cells (48.7%) which was associated with the loss of the mitochondrial potential (39.1%) as measured by CMXRos staining (Fig 2B and supplemental Fig. 1B). Apoptosis induction was also determined by propidium iodide (PI)-FACS analyses of fragmented nuclei, where FOXO3 activation by 4OHT increases the number of apoptotic cells to 31.1% and 52.1% apoptotic cells within 24 and 48 hours, respectively (Fig 2C). In Jurkat/FOXO3, Molt3/FOXO3 and Molt4/FOXO3 cells treatment with 4OHT induces cell death up to 27.4% (supplemental Fig. 1C). Apoptosis induction was efficiently blocked by the pan-caspase inhibitor qVD-OPH (10 µM), which reduced FOXO3-induced apoptosis from 31.3% to 3.7% after 24 hours and from 52.1 to 3.1% after 48 hours. This indicates that FOXO3 induces caspase-dependent cell death in T-ALL leukemia cells (Fig 2C).

### FOXO3 induces death regulators of the intrinsic and the extrinsic apoptosis signaling pathway

**Figure 3:**
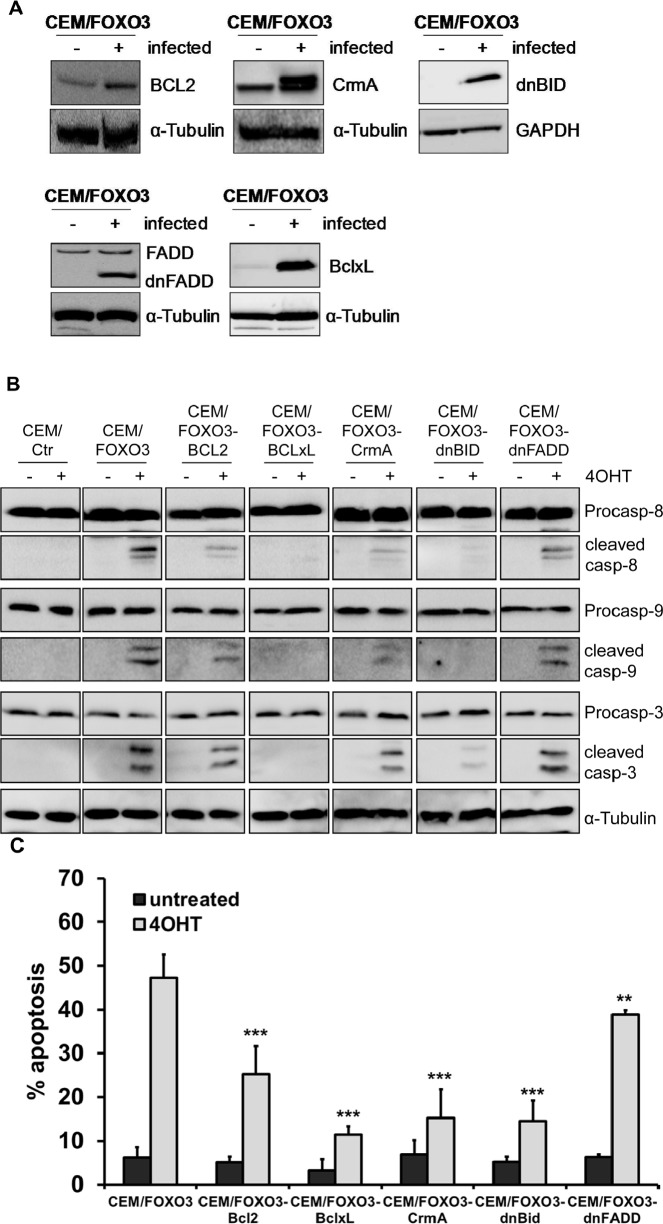
FOXO3-induced apoptosis depends on extrinsic and intrinsic death signaling CEM/FOXO3 cells were retrovirally infected with pLIB-BCL2-iresPuro, pLIB-CrmA-iresPuro, pLIB-dnFADD-iresPuro, pLIB-dnBID-iresPuro or pLIB-BclxL-iresPuro supernatants. Transgenic expression of BCL2, BclxL, CrmA, dnBID and dnFADD in these cells was verified by immunoblot analyses. Mock-infected cells were used as controls. α-Tubulin served as loading control (A). CEM/FOXO3-Ctr, CEM/FOXO3-BCL2, -BclxL, -CrmA, -dnBID, and -dnFADD cells were incubated with 4OHT (50 nM) for 18 hours. Cleavage of caspases-8, -9, and -3 was assessed by immunoblot analyses. α-Tubulin was used as loading control (B). Apoptosis induction was assessed by PI-FACS analyses (C). Statistical difference between 4OHT-treated controls and cell lines with ectopic expression of apoptosis inhibitors was calculated by unpaired t-test (***P < 0.001, **P < 0.005).

Depending on the cell type FOXO3 induces cell cycle arrest by elevating the expression of the cell cycle inhibitors p27^Kip1^ and/or p21^Cip1^ [23, 24] or triggers apoptosis. FOXO3 targets include death ligands of the extrinsic death machinery, like TRAIL or FASL [1, 19] or members controlling the intrinsic death pathway like Bim, Noxa or BIRC5/Survivin [17, 18, 25]. To identify the main signaling pathway underlying FOXO3-induced apoptosis in T-ALL cells, we generated CEM/FOXO3 cells overexpressing either anti-apoptotic members of the BCL2 family (BCL2, BclxL), inhibitors of the extrinsic death pathway (caspase-inhibitor CrmA, dominant-negative FADD) or a mutated version of the BH3-only protein BID (dnBID) that cannot be cleaved by caspases [26]. The stable ectopic expression of these proteins was analyzed by immunoblot (Fig 3A and supplemental Fig. 1D). We then assessed how inhibition of different steps of extrinsic and intrinsic apoptosis execution affects cleavage of caspase-8, -9, and -3 (Fig 3B). CEM/FOXO3 cells and sublines ectopically expressing BCL2, dnFADD, dnBID and CrmA were treated with 50 nM 4OHT for 18 hours and cell lysates were subjected to immunoblot analyses (Fig 3B). Whereas BCL2 and dnFADD only partially reduced cleavage of caspase-8, -9, and 3, CrmA and dnBID effectively lowered caspase-8, -9 and -3-processing and BclxL completely blocked cleavage of all three caspases, suggesting that apoptosis initiation most likely occurs upstream of mitochondria possibly involving additional, FADD-independent pathways.

To analyze how the inhibition of key-steps in extrinsic and intrinsic death signaling affects cell survival of T-ALL during FOXO3 activation, we measured the number of apoptotic cells by FACS-analyse of PI-stained nuclei. FOXO3 activation by 4OHT induced 47% apoptosis after 24 hours – the expression of CrmA (CEM/FOXO3-CrmA) and dnBID (CEM/FOXO3-dnBID) reduced the apoptosis rate to 15.2% and 14.5%, respectively. Overexpression of BclxL prevented apoptotic cell death almost completely (11.5%), whereas transgenic BCL2 lowered cell death to 25.3% and dnFADD reduced apoptosis by 10% (Fig 3C). Also in Jurkat/FOXO3, Molt3/FOXO3 and Molt4/FOXO3 cells, BclxL efficiently prevented FOXO3-induced apoptosis (supplemental Fig. 1E). These differences in the inhibitory effects of the various anti-apoptotic proteins can be explained by the weaker overexpression of the mutated FADD molecule compared to the endogenous FADD or by caspase-8 activation independent of DISC formation.

### TRAIL and Noxa are critical mediators of FOXO3-induced apoptosis in childhood T-ALL

**Figure 4:**
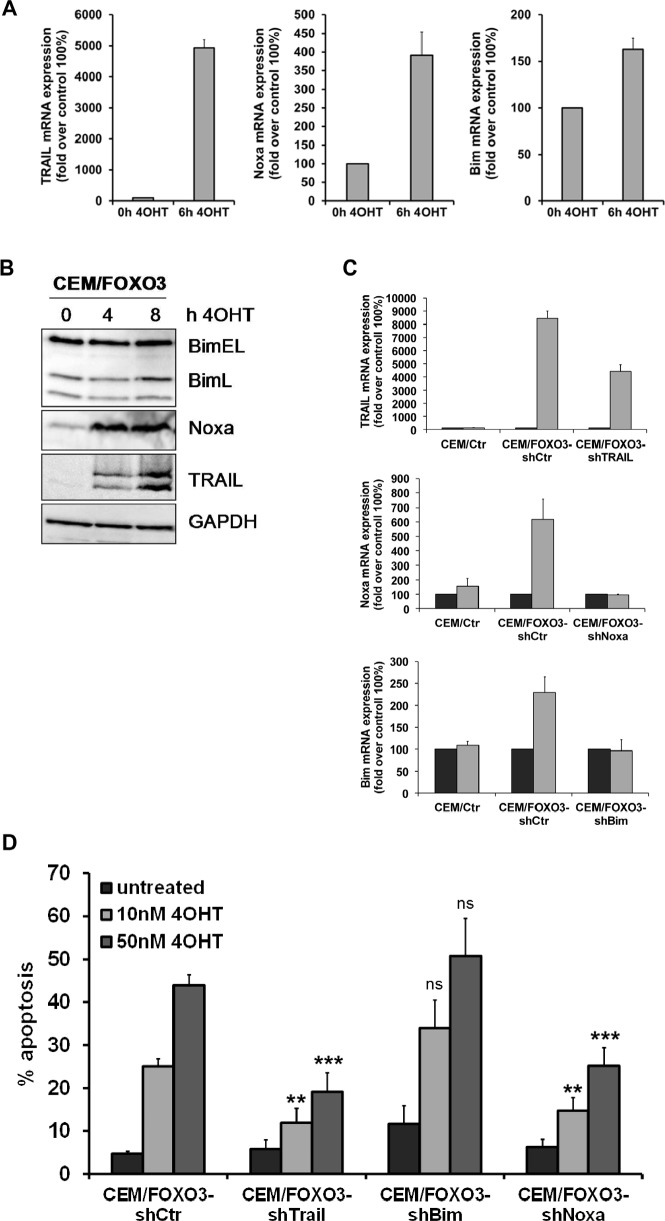
TRAIL and Noxa are critical mediators of FOXO3-induced apoptosis in T-ALL cells Total RNA was prepared from CEM/FOXO3 cells after incubation with 4OHT (50 nM) for 0 and 6 hours. The mRNA levels of TRAIL, Bim and Noxa were measured by quantitative RT-PCR (A). Induction of TRAIL, Bim and Noxa after incubation of CEM/FOXO3 cells with 4OHT (50 nM) for the times indicated was assessed by immunoblot analyses. Equal protein loading was confirmed by α-Tubulin (B). For knockdown experiments CEM/FOXO3 cells were infected with retroviruses coding for short-hairpin RNAs against TRAIL, Bim or Noxa. Knockdown efficiency was controlled by RT-PCR after incubation with 50 nM 4OHT for 4 hours (C). Apoptosis was measured by PI-FACS analyses in bulk-selected CEM/FOXO3-shCtr, -shTrail, -shBim or -shNoxa cells (D). Unpaired t-test was used to assess the statistical difference between 4OHT-treated controls and cell lines expressing shRNAs (***P < 0.001, **P < 0.005).

We next investigated potential FOXO3 targets that may be crucial for cell death initiation. We activated FOXO3 in CEM/FOXO3, Molt3/FOXO3 and Molt4/FOXO3 cells for 6 hours with 4OHT and analyzed the expression of several death inducers of the extrinsic and intrinsic cell death signaling pathways. Among them we identified TRAIL and the BH3-only proteins Bim and Noxa as FOXO3-regulated targets. TRAIL mRNA was induced up to 50fold after 6 hours, whereas e.g. FASL was not regulated (Fig 4A, left panel and supplemental Fig. 2), suggesting again extrinsic death receptor signaling. The BH3-only proteins Noxa and Bim where only slightly induced (4 fold and 1.6 fold, respectively) whereas other FOXO-targets such as Puma were not regulated at all (Fig 4A, middle and right panel and data not shown). The elevated expression of TRAIL, Noxa and Bim was also detectable on protein levels, although only the splice-variant BimL but not BimEL was induced after treatment with 4OHT (Fig 4B). To analyze whether the induction of TRAIL, Bim or Noxa was sufficient for cell death by FOXO3, we next performed knockdown experiments using stable expression of short-hairpin RNAs against TRAIL, Noxa or Bim. The efficiency of mRNA knockdown was verified by quantitative RT-PCR after treatment with 50 nM 4OHT for 4 hours (Fig 4C). Due to the strong induction of TRAIL by FOXO3 activation, TRAIL specific short-hairpin RNAs could not fully prevent TRAIL induction but reduced it to less than 50% of CEM/FOXO3-shCtr cells (Fig 4C, upper panel). The induction of Bim or Noxa by FOXO3 was almost completely inhibited by either Bim- or Noxa-specific short-hairpin RNAs (Fig 4C, middle and lower panel). PI-FACS analyses revealed that knockdown of TRAIL and Noxa efficiently reduced FOXO3-induced apoptosis, whereas knockdown of Bim did not reduce FOXO3-induced apoptosis. This observation suggests that FOXO3-induced apoptosis involves a concerted activation of extrinsic and intrinsic death signaling (Fig 4D), where Noxa acts as a mitochondrial sensitizer to apoptosis.

### Expression of p16INK4A represses FOXO3 and reduces dependency on the PI3K/PKB signaling

**Figure 5:**
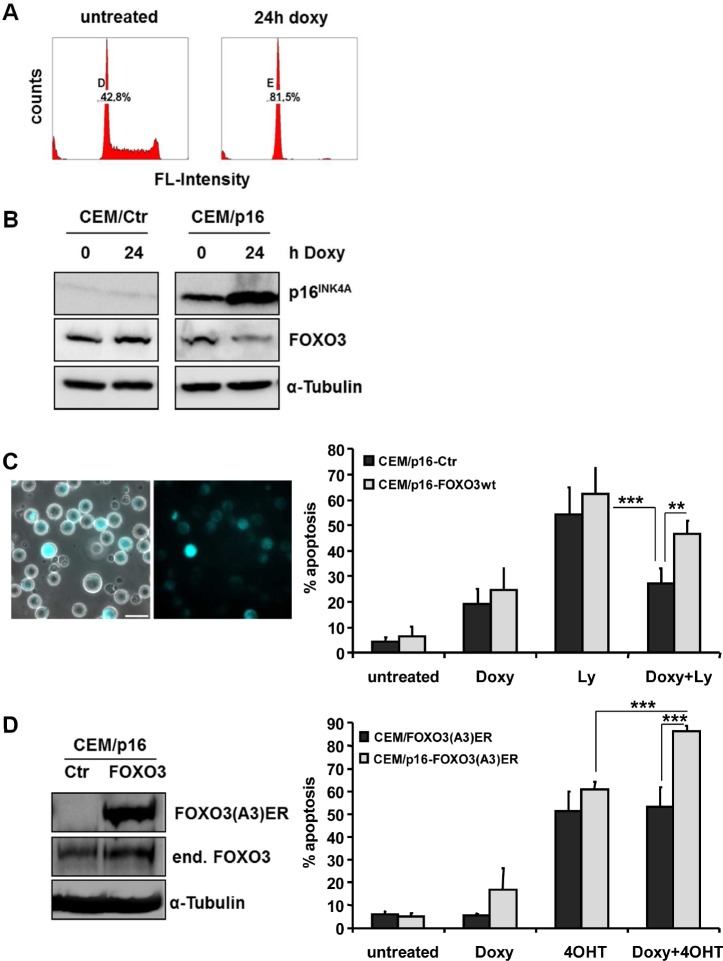
p16^INK4A^ regulates FOXO3 steady state expression and thereby apoptosis sensitivity CEM/p16 cells were treated for 24 hours with 250 ng/ml doxy. The G1-arrest was measured by flow cytometry after PI-staining (A). p16^INK4A^ and FOXO3 levels were assessed by immunoblot analysis after incubation of CEM/Ctr and CEM/p16 cells with doxy (250 ng/ml) for 24 hours. Equal protein loading was confirmed by α-Tubulin detection (B). CEM/Ctr, CEM/p16 and CEM/p16-ECFP-FOXO3wt cells were incubated with doxy for 24 hours and/or Ly294002 (40 µM) for another 48 hours. The expression of ECFP-FOXO3wt was assessed by live cell fluorescence microscopy. Bar is 50 µm (left panel). Apoptosis was assessed by PI-FACS analyses (C). CEM/p16 cells were retrovirally infected with pLIB-FOXO3(A3)ERtm-iresNeo supernatants. The expression of the fusion protein FOXO3(A3)ERtm and the endogenous FOXO3 were verified by immunoblot analyses. α-Tubulin was used as loading control (D, left panel). CEM/FOXO3-Ctr and CEM/p16-FOXO3(A3)ER cells were treated with doxy for 24 hours and/or 4OHT (50 nM) for another 36 hours (D, right panel). Apoptosis levels were assessed by PI-FACS analysis, for statistical analysis unpaired t-test was used (***P < 0.001, **P < 0.005).

We have shown before that loss of the INK4A gene changes the sensitivity of leukemia cells to certain apoptotic stimuli, such as glucocorticoid- or FAS-induced apoptosis [27, 28]. Re-introduction of p16^INK4A^ sensitizes leukemia cells to mitochondrial cell death by changing the balance of pro- and anti-apoptotic BCL2 proteins at the mitochondria [28]. Since FOXO3-induced cell death involves BH3-only proteins and causes loss of the mitochondrial potential (Fig 2B), we analyzed whether expression and activity of p16^INK4A^ and FOXO3 might be connected in ALL cells. As shown in Fig 5A the conditional reconstitution of p16^INK4A^ induces cell cycle arrest in the G1 phase and represses FOXO3 steady state expression levels within 24 hours (Fig 5B). This suggests that p16^INK4A^ reconstitution may shut down death sensitivity mediated by FOXO3 in T-ALL. Inhibition of the PI3K by the small compound inhibitor Ly294002 induces programmed cell death (54%, Fig 5C), which coincides with loss of PKB-phosphorylation at serine-473 (data not shown) and nuclear accumulation of FOXO3 (Supplemental Fig. 3A). The expression of p16^INK4A^ lowered Ly294002-induced cell death from 54% to 27%, suggesting that p16^INK4A^ expression reduces the PI3K survival signaling dependency of T-ALL cells (Fig 5C, right panel). To analyze whether altered FOXO3 expression is critical for this effect, we infected CEM/p16 cells with an ECFP-tagged wild-type FOXO3 construct to compensate for the reduction of endogenous FOXO3 during p16^INK4A^ expression. The expression of ECFP-FOXO3 was verified by live cell microscopy (Fig 5C, left panel). Ectopic expression of FOXO3 almost completely neutralized the protective effect of p16^INK4A^ and increased Ly294002-induced cell death from 27% to 47% in p16^INK4A^-expressing cells. To study whether p16^INK4A^ also modulates downstream signaling of FOXO3 in T-ALL cells, we infected CEM/p16 cells with the PKB-phosphorylation-independent FOXO3(A3)ER fusion protein and validated the expression by immunoblot (Fig 5D, left panel). To analyze apoptosis sensitivity CEM/FOXO3(A3)ER and CEM/p16-FOXO3(A3)ER cells were treated with 250 ng/ml doxycycline (doxy) for 24 hours and then the cells were treated with 50 nM 4OHT for another 36 hours to activate FOXO3. Combined treatment of CEM/p16-FOXO3(A3)ER cells with doxy and 4OHT increased cell death from 60.8% to 86.6%, implying that p16^INK4A^ expression accelerated FOXO3 apoptosis signaling. This is most likely due to the impact of p16^INK4A^ on the balance of pro- and anti-apoptotic BCL2 proteins at the mitochondria [28]. Therefore, p16^INK4A^ sensitizes T-ALL cells to apoptotic stimuli and in parallel efficiently represses FOXO3 steady state levels to reduce the detrimental effects of the pro-apoptotic FOXO3-targets Noxa and TRAIL.

**Figure 6:**
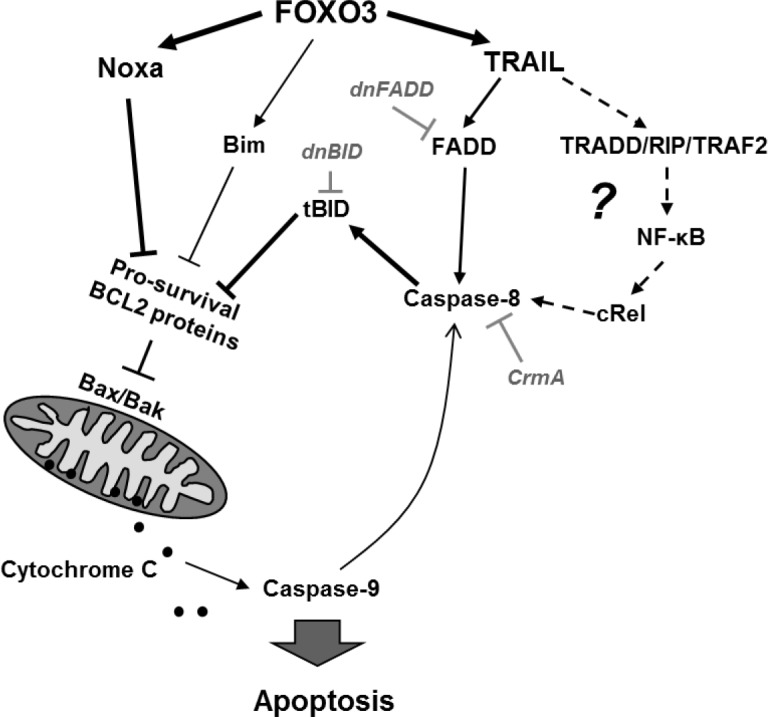
Model for FOXO3-induced apoptosis in T-ALL Our data suggest that the FOXO3 targets TRAIL and the BH3-only protein Noxa are critical for cell death induction. As dnFADD only partially inhibited FOXO3-induced cell death, an additional apoptosis signal might be triggered via TRADD/RIP/TRAF2 and NF-κB, leading then to cleavage of caspase-8 and activation of tBID independent of FADD. FOXO3-induced expression of Noxa on the other hand may partially sequester anti-apoptotic BCL2 proteins such as MCL1, BclxL and BCL2 and thereby sensitize mitochondria to tBID. The concerted impact of both apoptotic stimuli at mitochondria thereby will cause activation of BAX/BAK, loss of outer mitochondrial membrane integrity, Cytochrome *c* release and caspase-9 and -3 activation. Cleavage of caspase-8 by small amounts of caspase-9 may further amplify this death circuit eventually leading to Cytochrome *c* release and apoptosis.

## DISCUSSION

In the present study we demonstrate that activation of the transcription factor FOXO3 induces apoptotic cell death in therapy-resistant T-ALL cells. Leukemia cells frequently carry deletions of the phosphatase PTEN resulting in PKB hyperactivation [6] and as a consequence thereof FOXO3 inactivation and export from the nucleus. We found cytoplasmic localization of FOXO3 predominantly in the bone-marrow cells from patients who poorly respond to prednisone treatment (Fig 1). CEM T-ALL cells, which were used in this study, are a well-defined model for this leukemia subtype as they carry a homozygous deletion of the tumor suppressor PTEN [6] and were isolated from a patient after prednisone-therapy [29]. In these cells, the activation of a 4OHT-regulated, PKB-phosphorylation-independent FOXO3-transgene efficiently induces apoptosis (Fig 2A-C). We demonstrated that FOXO3 induces apoptosis via Bim and Noxa in neuroblastoma cells, where PKB signaling is also frequently deregulated [18]. Since T-ALL cells showed reduced DISC formation after extrinsic death signaling [22] we hypothesized that FOXO3 activation may involve components of the intrinsic death machinery. Ectopic expression of BclxL completely blocked FOXO3-induced apoptosis, supporting the model of mitochondria-induced cell death (Fig 3C), whereas BCL2 only partially rescued CEM cells from FOXO3-induced apoptosis. This may be caused by insufficient expression and/or stability of the transgene (Fig 3A) or the loss of high-expressing cells by BCL2-driven G1 arrest [30]. Dominant-negative FADD reduced FOXO3-induced apoptosis by 10%, which suggests that TRAIL may activate additional death-signaling pathways independent of FADD (Fig 3B). The caspase-8 inhibitor CrmA and dnBID not only completely blocked apoptosis, but also prevented the cleavage of caspase-9 (Fig 3B-C). This argues against a possible amplification loop via caspase-9 and caspase-8 after mitochondrial depolarization as the main cause of caspase-8 cleavage and suggests that TRAIL triggers caspase-8 cleavage at least in part FADD-independently. TRAIL can activate NF-κB signaling via recruitment of TRADD leading to either survival or apoptosis, which depends on activation of specific subunits of NF-κB [31, 32]. The strong induction of TRAIL after FOXO3 activation was also seen on mRNA and protein level (Fig 4A-B). The BH3-only protein Noxa was strongly induced by FOXO3 whereas Bim was only slightly elevated (Fig 4A-B). BH3-only proteins such as Bim, Noxa or tBID bind to anti-apoptotic BCL2 proteins such as BCL2 and BclxL and neutralize their pro-survival function [33]. Cleavage of BID after caspase-8 activation together with increased Noxa and Bim therefore might displace BAX and BAK from BCL2 and BclxL, leading to mitochondrial outer membrane pore-formation and mitochondrial damage. Noxa which is a weak BH3-only protein most likely sensitizes T-ALL cells to TRAIL signaling by inactivation of anti-apoptotic proteins. The induction of Noxa by FOXO3 is also observed in neuroblastoma cells [18] where it significantly contributes to mitochondrial cell death by releasing BAX and BAK from BclxL [34, 35]. Noxa knockdown efficiently prevented cell death in CEM cells (Fig 4D). This implies that Noxa acts in concert with truncated BID to destabilize the BCL2-rheostat and, together with TRAIL, mediates FOXO3-induced apoptosis in ALL cells.

Primary T-ALL cells frequently show loss of the INK4A gene locus and this genetic deletion also correlates with relapse [8, 36-38]. We have demonstrated before that reconstitution of this locus in CEM T-ALL cells leads to G1 arrest (Fig 5A) and sensitizes the leukemia cells to different apoptotic stimuli [27, 28]. Tetracyclin-regulated expression of p16^INK4A^ represses FOXO3 levels within 24 hours (Fig 5B), suggesting that as soon as the INK4A locus is deleted during leukemogenesis FOXO3 levels may rise and FOXO3 has to be functionally neutralized by hyperactive PKB. In this respect the development of T-ALL subclones with hyperactivated PI3K-PKB signaling due to e.g. deletion of the tumor suppressor PTEN might be forced by the loss of p16^INK4A^. In support of this hypothesis Ly294002 induces nuclear accumulation of FOXO3 and cell death (supplemental Fig. 3A-B) and p16^INK4A^-expressing T-ALL cells are less sensitive to inhibition of the PI3K by Ly294002 (Fig 5C). Stable expression of a FOXO3 wild-type protein (CEM/p16-ECFP-FOXO3wt cells) to compensate for the loss of endogenous FOXO3 abrogates the Ly294002-protective effect of p16^INK4A^ and restores apoptosis sensitivity to the level of non-p16^INK4A^-expressing cells (Fig 5C). This proves that the repression of FOXO3 by p16^INK4A^ reduces Ly294002 sensitivity. Interestingly if a PKB-phosphorylation-independent FOXO3(A3)ER allele is activated by 4OHT in p16^INK4A^-expressing cells, p16^INK4A^ further increases cell death (Fig 5D). These results are consistent with our earlier report on p16^INK4A^-induced changes of the balance of pro- and anti-apoptotic BCL2 proteins [28]. As p16^INK4A^ causes repression of the Noxa-binding partner MCL1 and in parallel increases Puma and decreases BCL2-expression, p16^INK4A^-expressing cells will be much more sensitive to induction of Noxa and activation of BID via TRAIL by FOXO3. Therefore the repression of endogenous FOXO3 by p16^INK4A^ is even essential for p16^INK4A^-expressing cells as the disturbed balance of pro-and anti-apoptotic BCL2 proteins at mitochondria renders these cells highly sensitive to the detrimental effects of the FOXO3-targets Noxa and TRAIL.

Our results demonstrate for the first time a link between loss of p16^INK4A^ and FOXO3-inactivation / hyperactive PKB-signaling in T-ALL and both cancer-associated alterations correlate with therapy resistance in leukemia cells. Both proteins affect the balance between pro- and anti-apoptotic BCL2-proteins and thereby determine drug sensitivity at the level of mitochondria. Thereby, FOXO3 provides an interesting and promising target in the PI3K-PKB pathway for cancer therapy, as its posttranslational inactivation causes apoptosis resistance.

## MATERIAL AND METHODS

### Cell lines, culture conditions and reagents

Jurkat, Molt3, Molt4, CEM/C7H2 (CEM), a subclone of the CCRF/CEM-C7 cell line and all transgenic variants were cultured in RPMI 1640 (BioWhittaker, Belgium) containing 5% fetal calf serum (FCS; Gibco BRL, Paisley, GB), 100 U/ml penicillin, 100 µg/ml streptomycin and 2 mM L-glutamine (PAA, Pasching Austria) at 5% CO_2_ and 37 °C in saturated humidity. Generation and analysis of the p16^INK4A^-expressing clone 6E2/p16 have been described before [27]. Phoenix™ packaging cells for production of amphotropic retroviruses (kindly provided by G. Nolan, Standford) were cultured in RPMI (BioWhittaker, Belgium), containing 10% fetal calf serum. Ly294002 was obtained from Cayman Chemical Europe (Talinn, Estonia). All cultures were routinely tested for mycoplasma contamination (Venor^R^GeM-mycoplasma detection kit, Minerva). All reagents were purchased from Sigma-Aldrich (Vienna, Austria) unless indicated otherwise. For each experiment, mid-log phase cultures were seeded in fresh medium.

### Immunofluorescence

Primary T-ALL cell culture were placed on the slide by cytospin and then fixed with formaldehyde (4%) for 15 min. Slides were treated with NH_4_Cl (Sigma-Aldrich) 50 mM for 15 min to reduce background.

Cells were permeabilized with 0.1% Triton X-100/phosphate buffered saline for 3 min and incubated first with blocking buffer (5% bovine serum albumin in phosphate-buffered saline) and then with monoclonal FOXO3 antibody (1:100, Cell Signaling Technology Inc., Boston, USA) diluted in blocking buffer overnight at 4 °C. Cells were then incubated for 1 h with specific secondary antibody conjugated to Alexa Fluor 488 (1:1000; Invitrogen, Carlsbad CA, USA), and for 10 min with 4.6-diamidino-2-phenylindole (DAPI) 1:10000; Sigma-Aldrich, Vienna, Austria). Images were acquired by the videoconfocal system ViCo microscope Nikon Eclipse 80i (Nikon, Japan).

### Primary cell cultures

T-ALL patient samples were obtained after informed consent following the tenets of the Declaration of Helsinki. Diagnosis was made according to standard cytomorphology, cytochemistry and immunophenotypic criteria. All analyzed T-ALL samples were obtained at the time of diagnosis before treatment, after Ficoll–Hypaque (Pharmacia, Uppsala, Sweden) separation of mononuclear cells. Mononuclear cells were frozen as viable cells in FCS and 10% DMSO and stored in liquid nitrogen. The percentage of CD7 cells ranged from 85 to 96%.

### Production of retroviruses and retroviral infection

Approximately 6x10^5^ Phoenix™ packaging cells were transfected with 2 µg of retroviral vector and 1 µg of a plasmid coding for VSV-G protein using Lipofectamine2000 (Invitrogen, Carlsbad CA, USA). Following vectors have been described previously: pLIB-FOXO3(A3)ERtm-iresNeo, pLIB-BCL2-iresPuro, pLIB-BclxL-iresPuro, pLIB-CrmA-iresPuro, pLIB-dnBID-iresPuro, pLIB-dnFADD-iresPuro, pQ-tetH1-shBim-SV40-Puro, pQ-tetH1-shNoxa-SV40Puro, pLIB-ECFP-FOXO3wt-iresPuro, pLIB-MCS2-iresPuro, pLIB-MCS2-iresNeo and pQ-tetH1-SV40Puro [18, 28, 34, 35]. For specific gene knockdown of TRAIL annealed oligonucleotides coding for the TRAIL-specific shRNA sequence GCAGATGCAGGACAAGTACT were inserted into the *BamH1-Mun1* sites of the plasmid pQ-tetH1-SV40-Puro. Plasmids were transfected into Phoenix packaging cells to generate supernatants. After 48h, the retrovirus-containing supernatants were filtered (0.22µm syringe filters, Sartorius, Germany) and used to infect CEM cells. The resulting cell lines are: CEM/FOXO3-Ctr, CEM/FOXO3-BCL2, CEM/FOXO3-BclxL, CEM/FOXO3-CrmA, CEM/FOXO3-dnFADD, CEM/FOXO3-dnBID, CEM/FOXO3-shCtr, CEM/FOXO3-shBim, CEM/FOXO3-shNoxa, CEM/FOXO3-shTRAIL, CEM/p16-ECFP-FOXO3wt and CEM/p16-FOXO3(A3)ER.

### Detection of apoptosis

Apoptosis was assessed by staining the cells with propidium-iodide (PI) or AnnexinV using a CytomicsFC-500 Beckman Coulter as previously described [39]: 2x10^5^ cells were centrifuged and resuspended in PI solution containing 0.1% Triton X-100. Stained nuclei in the sub-G1 region were considered to represent apoptotic cells. Mitochondrial activity and membrane potential were assessed by the fluorescence dye MitoTracker Red/CMX-Ros (Invitrogen, USA). For measurement of AnnexinV positive cells 5x10^5^ cells were harvested and stained with FITC-labeled AnnexinV (Alexis Biochemicals, San Diego, CA, USA) in Annexin Binding Buffer.

### Immunoblot

5x10^6^ cells were lysed on ice in lysis-buffer (50 mM HEPES/NaOH, 1% Triton X-100, 150 mM NaCl, 2 mM EDTA, 10% glycerol) with protease and phosphatase inhibitors. Protein concentration was measured using Bradford-Reagent (BioRad Laboratories, Munich, Germany). Equal amounts of total protein (50 μg/lane) were separated by SDS-PAGE and transferred to nitrocellulose membranes (Schleicher & Schuell, Germany) by a semi-dry blotting device (Hoefer TE70, Amersham Biosciences). Membrane blocking was performed with PBS blocking buffer containing 0.1% Tween20 and 5% nonfat dry milk, incubated with primary antibodies specific for human BclxL, caspase-3, BID, FOXO3, (Cell Signaling Technology Inc., Boston, USA), BCL2, Bim, CrmA, caspase-8, FADD (BD Biosciences - Pharmingen, Heidelberg, D), Noxa (Alexis Corporation, Lausen, CH), TRAIL (Acris, Herford, Germany), caspase-9 (R&D Systems, Minneapolis, MN, USA) and α-Tubulin (Oncogene Research Products, USA) then washed and incubated with anti-mouse, anti-rabbit or anti-rat horseradish-peroxidase-conjugated secondary antibodies (GE Healthcare, USA). The immunoblots were developed by enhanced chemiluminescence (GE Healthcare, USA) according to the manufacturer´s instructions and analyzed in an AutoChemi detection system (UVP, England).

### Quantitative real-time RT-PCR

To quantify Bim, Noxa, and TRAIL mRNA levels we designed real-time RT-PCR assays, using GAPDH as reference gene. Total RNA was isolated from 5x10^6^ cells using TRIzol™ Reagent (Sigma-Aldrich, Vienna, Austria) according to the manufacturer´s instructions. Complementary DNA was synthesized from 1 µg of total RNA using the RevertAid™ First Strand H minus cDNA Synthesis Kit (Thermo Scientific, Sankt Leon-Rot, Germany). The oligonucleotides to amplify mRNA fragments were Bim (forward 5'-AGCACCCATGAGTTGTGACAAATC, reverse 3'-CGTTAAACTCGTCTCCAATACGC), Noxa (forward 5'-AGCAGAGCTGGAAGTCGAGTGTG, reverse 3'-TGATGCAGTCAGGTTCCTGAGC), TRAIL (forward 5'-AAAGAGGTCCTCAGAGAGTAGCAGC, reverse 3'-GCTCAGGAATGAATGCCCACTC), and GAPDH (forward 5'-TGTTCGTCATGGGTGTGAACC, reverse 3'-GCAGTGATGGCATGGACTGTG) and were synthesized by Microsynth AG (Balgach, Switzerland). After normalization on GAPDH expression, regulation was calculated between treated and untreated cells. Control is set as 100% expression.

### Statistics

Statistical significance of differences between controls and treated cells were calculated using unpaired t-test. All statistical analyses were performed using Graph Pad Prism 4.0 software.

## SUPPLEMENTARY FIGURES



## References

[1] Brunet A, Bonni A, Zigmond MJ, Lin MZ, Juo P, Hu LS, Anderson MJ, Arden KC, Blenis J, Greenberg ME (1999). Akt promotes cell survival by phosphorylating and inhibiting a Forkhead transcription factor. Cell.

[2] Zhang J, Grindley JC, Yin T, Jayasinghe S, He XC, Ross JT, Haug JS, Rupp D, Porter-Westpfahl KS, Wiedemann LM, Wu H, Li L (2006). PTEN maintains haematopoietic stem cells and acts in lineage choice and leukaemia prevention. Nature.

[3] Arden KC (2006). Multiple roles of FOXO transcription factors in mammalian cells point to multiple roles in cancer. Experimental Gerontology.

[4] Zhao WL (2009). Targeted therapy in T-cell malignancies: dysregulation of the cellular signaling pathways. Leukemia.

[5] Martelli AM, Nyakern M, Tabellini G, Bortul R, Tazzari PL, Evangelisti C, Cocco L (2006). Phosphoinositide 3-kinase//Akt signaling pathway and its therapeutical implications for human acute myeloid leukemia. Leukemia.

[6] Sakai A, Thieblemont C, Wellmann A, Jaffe ES, Raffeld M (1998). PTEN gene alterations in lymphoid neoplasms. Blood.

[7] Mimeault M, Batra SK (2009). Recent insights into the molecular mechanisms involved in aging and the malignant transformation of adult stem/progenitor cells and their therapeutic implications. Ageing Res.Rev.

[8] Yilmaz OH, Valdez R, Theisen BK, Guo W, Ferguson DO, Wu H, Morrison SJ (2006). Pten dependence distinguishes haematopoietic stem cells from leukaemia-initiating cells. Nature.

[9] Jotta PY, Ganazza MA, Silva A, Viana MB, da Silva MJ, Zambaldi LJG, Barata JT, Brandalise SR, Yunes JA (2009). Negative prognostic impact of PTEN mutation in pediatric T-cell acute lymphoblastic leukemia. Leukemia.

[10] Markman B, Dienstmann R, Tabernero J (2010). Targeting the PI3K/Akt/mTOR Pathway GÇô Beyond Rapalogs. Oncotarget.

[11] Miyamoto K, Araki KY, Naka K, Arai F, Takubo K, Yamazaki S, Matsuoka S, Miyamoto T, Ito K, Ohmura M, Chen C, Hosokawa K, Nakauchi H, Nakayama K, Nakayama KI, Harada M, Motoyama N, Suda T, Hirao A (2007). Foxo3a is essential for maintenance of the hematopoietic stem cell pool. Cell Stem Cell.

[12] Tothova Z, Kollipara R, Huntly BJ, Lee BH, Castrillon DH, Cullen DE, McDowell EP, Lazo-Kallanian S, Williams IR, Sears C, Armstrong SA, Passegue E, DePinho RA, Gilliland DG (2007). FoxOs are critical mediators of hematopoietic stem cell resistance to physiologic oxidative stress. Cell.

[13] Andreu EJ, Lledo E, Poch E, Ivorra C, Albero MP, Martinez-Climent JA, Montiel-Duarte C, Rifon J, Perez-Calvo J, Arbona C, Prosper F, Porez-Roger I (2005). BCR-ABL Induces the Expression of Skp2 through the PI3K Pathway to Promote p27Kip1 Degradation and Proliferation of Chronic Myelogenous Leukemia Cells. Cancer Research.

[14] Essafi A, Fernandez de Mattos S, Hassen YAM, Soeiro I, Mufti GJ, Thomas NS, Medema RH, Lam EWF (2005). Direct transcriptional regulation of Bim by FoxO3a mediates STI571-induced apoptosis in Bcr-Abl-expressing. cells.

[15] Gu TL, Tothova Z, Scheijen B, Griffin JD, Gilliland DG, Sternberg DW (2004). NPM-ALK fusion kinase of anaplastic large-cell lymphoma regulates survival and proliferative signaling through modulation of FOXO3a. Blood.

[16] Paik JH, Kollipara R, Chu G, Ji H, Xiao Y, Ding Z, Miao L, Tothova Z, Horner JW, Carrasco DR, Jiang S, Gilliland DG, Chin L, Wong WH, Castrillon DH, DePinho RA (2007). FoxOs are lineage-restricted redundant tumor suppressors and regulate endothelial cell homeostasis. Cell.

[17] Dijkers PF, Medema RH, Lammers JWJ, Koenderman L, Coffer PJ (2000). Expression of the pro-apoptotic Bcl-2 family member Bim is regulated by the forkhead transcription factor FKHR-L1. Current Biology.

[18] Obexer P, Geiger K, Ambros PF, Meister B, Ausserlechner MJ (2007). FKHRL1-mediated expression of Noxa and Bim induces apoptosis via the mitochondria in neuroblastoma cells. Cell Death Differ.

[19] Modur V, Nagarajan R, Evers BM, Milbrandt J (2002). FOXO proteins regulate tumor necrosis factor-related apoptosis inducing ligand expression. Implications for PTEN mutation in prostate cancer. J.Biol.Chem.

[20] Tang TT, Dowbenko D, Jackson A, Toney L, Lewin DA, Dent AL, Lasky LA (2002). The forkhead transcription factor AFX activates apoptosis by induction of the BCL-6 transcriptional repressor. J.Biol.Chem.

[21] Letai A, Bassik MC, Walensky LD, Sorcinelli MD, Weiler S, Korsmeyer SJ (2002). Distinct BH3 domains either sensitize or activate mitochondrial apoptosis. serving as prototype cancer therapeutics, Cancer Cell.

[22] Scaffidi C, Fulda S, Srinivasan A, Friesen C, Li F, Tomaselli KJ, Debatin KM, Krammer PH, Peter ME (1998). Two CD95 (APO-1/Fas) signaling pathways. EMBO J.

[23] Kops GJ, Medema RH, Glassford J, Essers MA, Dijkers PF, Coffer PJ, Lam EW, Burgering BM (2002). Control of cell cycle exit and entry by protein kinase B-regulated forkhead transcription factors. Mol.Cell Biol.

[24] Seoane J, Le HV, Shen L, Anderson SA, Massague J (2004). Integration of Smad and forkhead pathways in the control of neuroepithelial and glioblastoma cell proliferation. Cell.

[25] Obexer P, Hagenbuchner J, Unterkircher T, Sachsenmaier N, Seifarth C, Bock G, Porto V, Geiger K, Ausserlechner M (2009). Repression of BIRC5/survivin by FOXO3/FKHRL1 sensitizes human neuroblastoma cells to DNA damage-induced apoptosis. Mol.Biol.Cell.

[26] Werner AB, de Vries E, Tait SW, Bontjer I, Borst J (2002). TRAIL receptor and CD95 signal to mitochondria via FADD, caspase-8/10, Bid, and Bax but differentially regulate events downstream from truncated Bid. J.Biol.Chem.

[27] Ausserlechner MJ, Obexer P, Wiegers GJ, Hartmann BL, Geley S, Kofler R (2001). The cell cycle inhibitor p16(INK4A) sensitizes lymphoblastic leukemia cells to apoptosis by physiologic glucocorticoid levels. J.Biol.Chem.

[28] Obexer P, Hagenbuchner J, Rupp M, Salvador C, Holzner M, Deutsch M, Porto V, Kofler R, Unterkircher T, Ausserlechner MJ (2009). p16INK4A sensitizes human leukemia cells to FAS- and glucocorticoid-induced apoptosis via induction of BBC3/Puma and repression of MCL1 and BCL2. J.Biol.Chem.

[29] Foley GE, Lazarus H, Farber S, Uzman BF, Boone BA, McCarthy RE (1965). Continuous culture of human lymphoblasts from peripheral blood of a child with acute leukemia. Cancer.

[30] Janumyan YM, Sansam CG, Chattopadhyay A, Cheng NL, Soucie EL, Penn LZ, Andrews D, Knudson CM, Yang E (2003). Bcl-x(L)/Bcl-2 coordinately regulates apoptosis. cell cycle arrest and cell cycle entry, Embo Journal.

[31] Shetty S, Gladden JB, Henson ES, Hu X, Villanueva J, Haney N, Gibson SB (2002). Tumor necrosis factor-related apoptosis inducing ligand (TRAIL) up-regulates death receptor 5 (DR5) mediated by NF kappa B activation in epithelial derived cell lines. Apoptosis.

[32] Chen X, Kandasamy K, Srivastava RK (2003). Differential Roles of RelA (p65) and c-Rel Subunits of Nuclear Factor +¦B in Tumor Necrosis Factor-related Apoptosis-inducing Ligand Signaling. Cancer Research.

[33] Labi V, Grespi F, Baumgartner F, Villunger A (2008). Targeting the Bcl-2-regulated apoptosis pathway by BH3 mimetics: a breakthrough in anticancer therapy?. Cell Death and Differentiation.

[34] Hagenbuchner J, Ausserlechner MJ, Porto V, David R, Meister B, Bodner M, Villunger A, Geiger K, Obexer P (2010). The Antiapoptotic Protein BCL2L1/BCL-XL is Neutralized by Proapoptotic PMAIP1/Noxa in Neuroblastoma Thereby Determining Bortezomib-Sensitivity Independent of Prosurvival MCL1 Expression. J.Biol.Chem.

[35] Hagenbuchner J, Kuznetsov AV, Hermann M, Hausott B, Obexer P, Ausserlechner MJ (2012). FOXO3-induced reactive oxygen species are regulated by BCL2L11/Bim and SESN3. J.Cell Sci.

[36] Fizzotti M, Cimino G, Pisegna S, Alimena G, Quartarone C, Mandelli F, Pelicci PG, Lo CF (1995). Detection of homozygous deletions of the cyclin-dependent kinase 4 inhibitor (p16) gene in acute lymphoblastic leukemia and association with adverse prognostic features. Blood.

[37] Kees UR, Burton PR, Lu C, Baker DL (1997). Homozygous deletion of the p16/MTS1 gene in pediatric acute lymphoblastic leukemia is associated with unfavorable clinical outcome. Blood.

[38] Okuda T, Shurtleff SA, Valentine MB, Raimondi SC, Head DR, Behm F, Curcio-Brint AM, Liu Q, Pui CH, Sherr CJ (1995). Frequent deletion of p16INK4a/MTS1 and p15INK4b/MTS2 in pediatric acute lymphoblastic leukemia. Blood.

[39] Hagenbuchner J, Kuznetsov AV, Obexer P, Ausserlechner MJ (2012). BIRC5/Survivin enhances aerobic glycolysis and drug resistance by altered regulation of the mitochondrial fusion/fission machinery. Oncogene.

