# Supplementary material for: Therapy-resistant acute lymphoblastic leukemia (ALL) cells inactivate FOXO3 to escape apoptosis induction by TRAIL and Noxa
Source: Oncotarget. 2013 Jun 25;4(7):995–1007. doi: 10.18632/oncotarget.953 (PMC3759677; doi:10.18632/oncotarget.953)
Supplement: Supplementary file 1 [file oncotarget-04-995-s001.pdf]

Therapy-resistant acute lymphoblastic leukemia (ALL) cells inactivate FOXO3 to escape apoptosis induction by TRAIL and Noxa - Ausserlechner et al

A

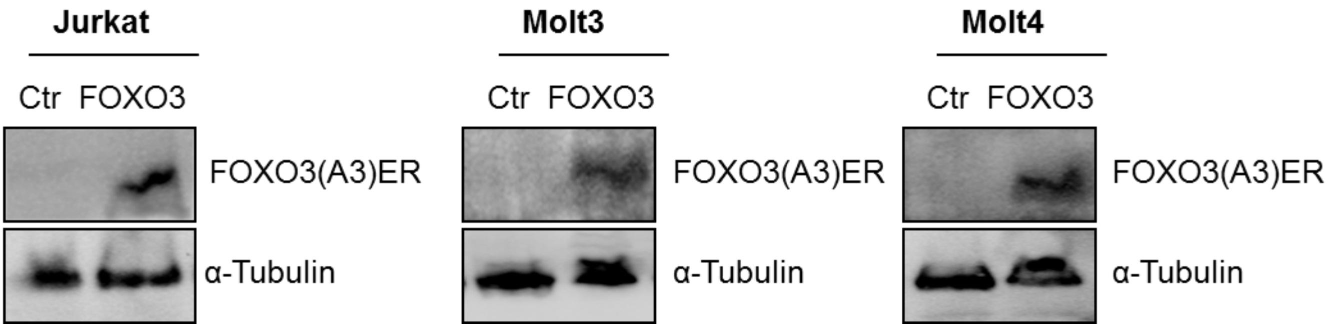

B

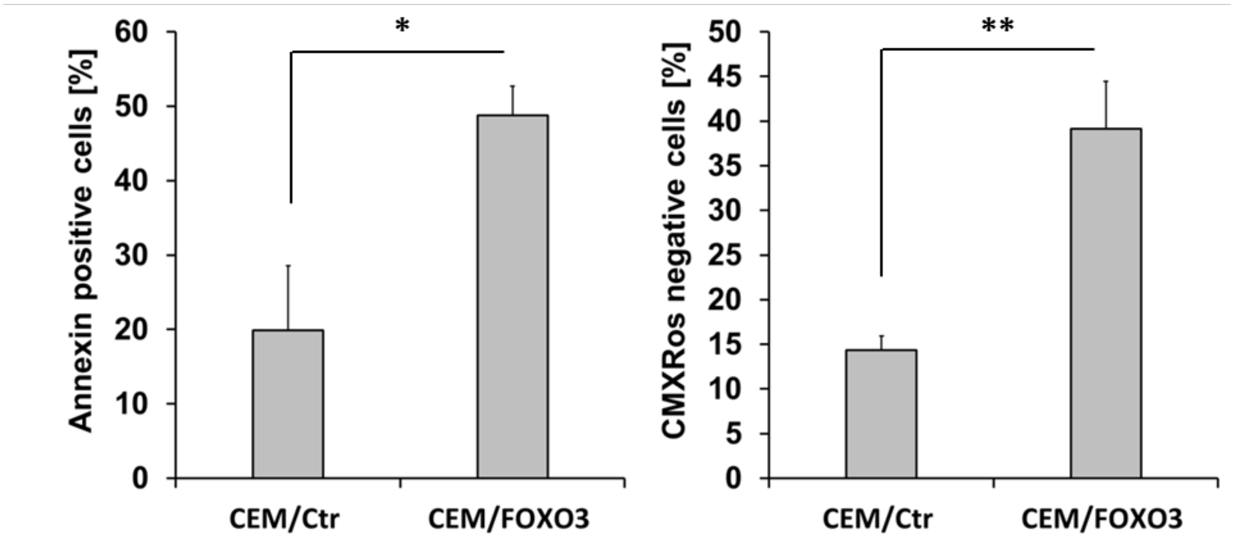

Suppl. Fig. 1: The T-ALL cell lines Jurkat, Molt3 and Molt4 were infected with the pLIB-FOXO3(A3)ERtm-Neo construct. The expression of the FOXO3 transgene was verified by immunoblot (A). CEM/FOXO3 cells were incubated with 4OHT (50 nM) for 24 hours to activate transgenic FOXO3. Exposure of phosphatidylserin was analyzed by AnnexinV staining and loss of mitochondrial activity was detected by CMXRos staining. Shown is the mean of three independent experiments. Statistical difference between treatments was assessed by unpaired t-test (*P <0.017;**P <0.004) (B).

C

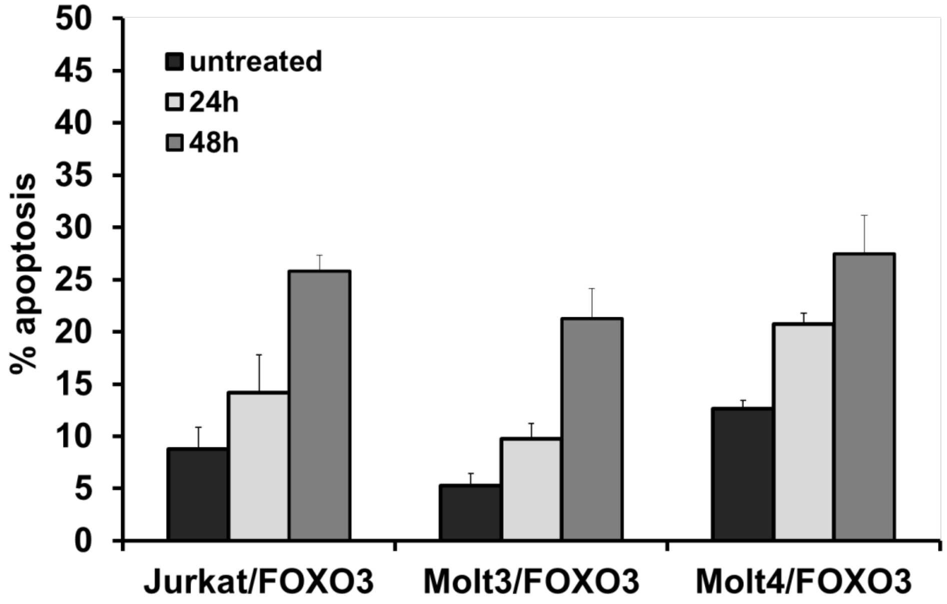

D

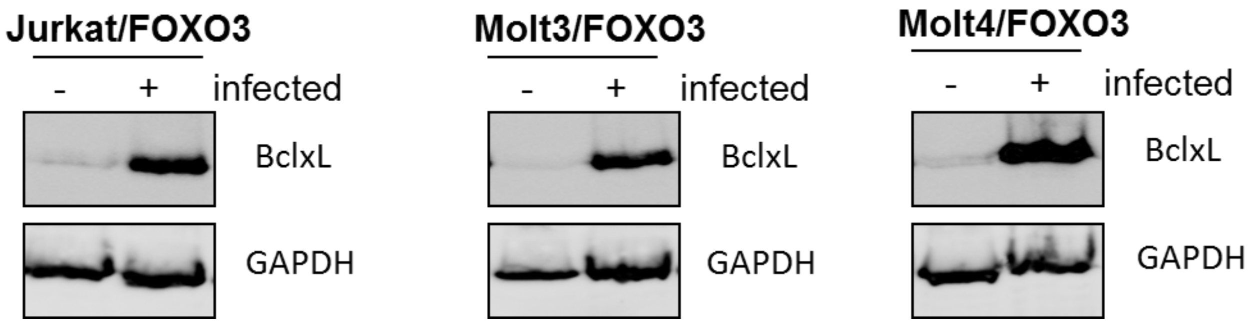

Suppl. Fig. 1: PI-FACS analyses of Jurkat/FOXO3, Molt3/FOXO3 and Molt4/FOXO3 cells after treatment with 100 nM 4OHT for up to 48 hours. Shown is the mean of three independent experiments (C). The cell lines Jurkat/FOXO3, Molt3/FOXO3, and Molt4/FOXO3 were infected with a retrovirus coding for BclxL. The expression of the transgene was verified by immunoblot, GAPDH served as loading control (D).

E

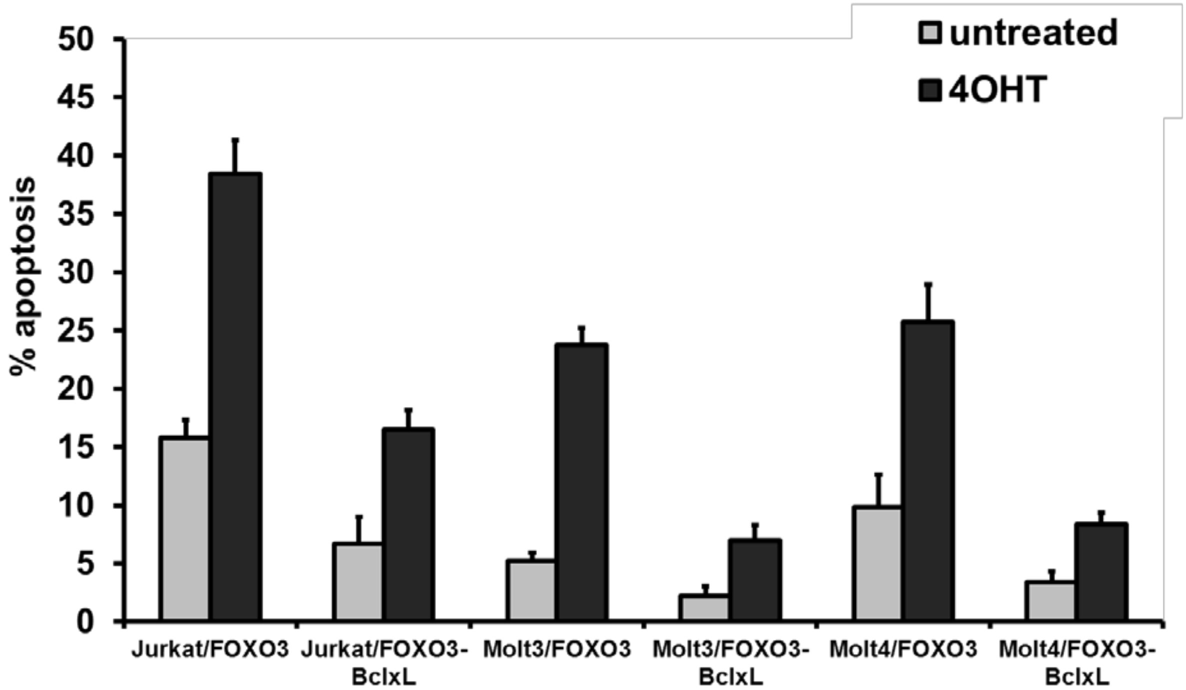

Suppl. Fig. 1: Effect of BclxL on FOXO3-induced cell death in different T-ALL cell lines. BclxL-overexpressing T-ALL cell lines and mock-infected controls were subjected to PI-FACS analyses after treatment with 100 nM 4OHT for 48 hours. Shown is the mean of four independent experiments (E).

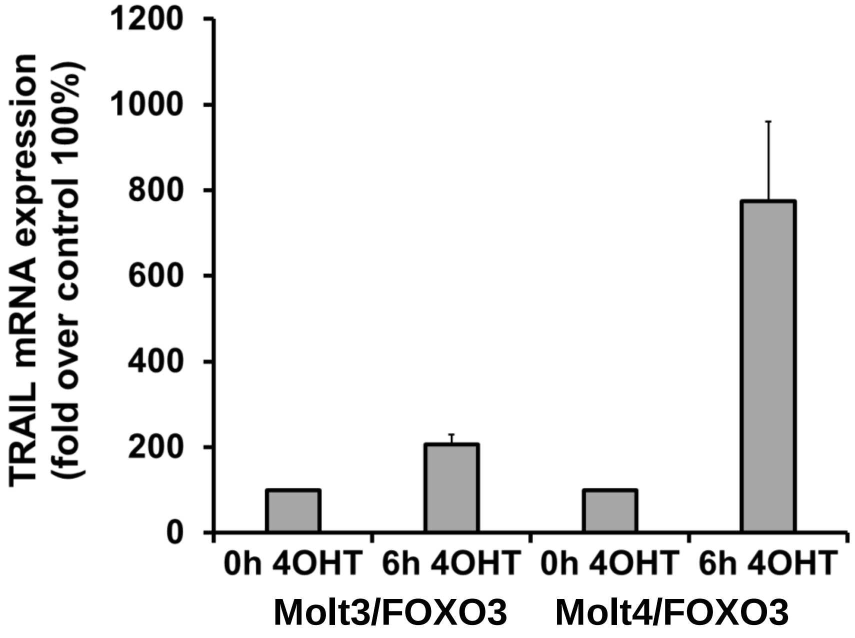

Suppl. Fig. 2: The T-ALL cells lines Molt3/FOXO3 and Molt4/FOXO3 were treated for 6 hours with 100 nM 4OHT. The mRNA level of TRAIL was analyzed by quantitative RT-PCR. Shown is the mean of three independent experiments each performed in triplicates.

A

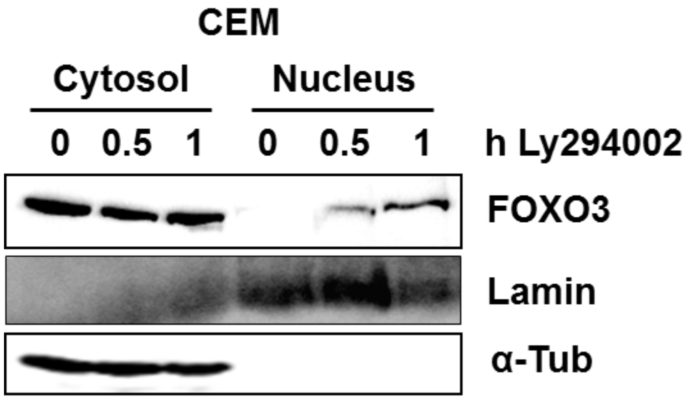

B

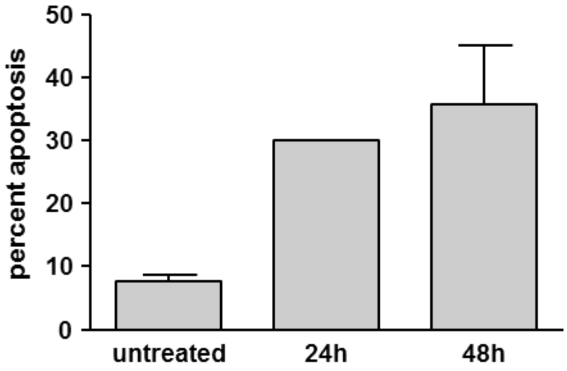

Suppl. Fig. 3: CEM-T-ALL cells were treated with 40 µM Ly294002 for up to one hour and then nuclear and cytoplasmic extracts were prepared by gradient centrifugation according to manufacturer's instruction (ProteoJet Kit, ThermoScientific, Sankt Leon-Rot, Germany). Endogenous FOXO3 expression was determined by immunoblot. LaminA/C (Cell Signaling, Boston, US) and α-tubulin (cytosolic) served as controls for the purity of nuclear and cytosolic fractions, respectively (A). CEM cells were treated for 24 and 48 hours with 40 µM Ly294002 and propidium-iodide stained nuclei were analyzed by flow cytometry in a FC500 flow cytometer (B).
